# Placental Toll-Like Receptor 3 and Toll-Like Receptor 7/8 Activation Contributes to Preeclampsia in Humans and Mice

**DOI:** 10.1371/journal.pone.0041884

**Published:** 2012-07-27

**Authors:** Piyali Chatterjee, Laura E. Weaver, Karen M. Doersch, Shelley E. Kopriva, Valorie L. Chiasson, Samantha J. Allen, Ajay M. Narayanan, Kristina J. Young, Kathleen A. Jones, Thomas J. Kuehl, Brett M. Mitchell

**Affiliations:** 1 Department of Internal Medicine, Texas A&M Health Science Center/Scott and White Memorial Hospital, Temple, Texas, United States of America; 2 Department of Pathology, Texas A&M Health Science Center/Scott and White Memorial Hospital, Temple, Texas, United States of America; 3 Department of Obstetrics and Gynecology, Texas A&M Health Science Center/Scott and White Memorial Hospital, Temple, Texas, United States of America; Lund University Hospital, Sweden

## Abstract

Preeclampsia (PE) is a pregnancy-specific hypertensive syndrome characterized by excessive maternal immune system activation, inflammation, and endothelial dysfunction. Toll-like receptor (TLR) 3 activation by double-stranded RNA (dsRNA) and TLR7/8 activation by single-stranded RNA (ssRNA) expressed by viruses and/or released from necrotic cells initiates a pro-inflammatory immune response; however it is unknown whether viral/endogenous RNA is a key initiating signal that contributes to the development of PE. We hypothesized that TLR3/7/8 activation will be evident in placentas of women with PE, and sufficient to induce PE-like symptoms in mice. Placental immunoreactivity and mRNA levels of TLR3, TLR7, and TLR8 were increased significantly in women with PE compared to normotensive women. Treatment of human trophoblasts with the TLR3 agonist polyinosine-polycytidylic acid (poly I:C), the TLR7-specific agonist imiquimod (R-837), or the TLR7/8 agonist CLO97 significantly increased TLR3/7/8 levels. Treatment of mice with poly I:C, R-837, or CLO97 caused pregnancy-dependent hypertension, endothelial dysfunction, splenomegaly, and placental inflammation. These data demonstrate that RNA-mediated activation of TLR3 and TLR7/8 plays a key role in the development of PE.

## Introduction

Preeclampsia (PE), a condition which affects ∼10% of pregnancies, can lead to significant morbidity and mortality in both the mother and the fetus and can also increase the risk of future cardiovascular disease in the mother [1]–[4]. PE is defined as hypertension and proteinuria at or after 20 weeks gestation and is associated with endothelial dysfunction and premature birth. The placenta is a key player in the etiology of PE as symptoms typically abate after placental delivery.

While there are many theories regarding the causes of PE, the exact origins of the disorder remain elusive. Proposed causes include inadequate trophoblast invasion, angiogenic imbalance, and improper adaptation of the immune system to the state of pregnancy [5], [6]. During pregnancy, the immune system must tolerate a half-foreign fetus, while still defending the body from pathogens. Many groups have hypothesized that an inadequate tolerance of the fetus by the maternal immune system may manifest as PE [7]–[10]. This hypothesis is strengthened by the fact that pregnant women who had a previous pregnancy with the same partner are less likely to develop PE than nulliparous women or women conceiving for the first time with a new partner. This effect is presumably due to a protective effect by previous contact with antigens [11]–[13]. However, the molecular mechanism by which the immune system contributes to the etiology of PE remains undefined.

The immune system responds to not only foreign pathogens but also endogenous markers of cellular damage. These “danger signals” or pathogen-associated molecular patterns (PAMPs) include RNA, DNA, heat shock proteins, uric acid, tumor necrosis factor, and others [6]. Sources of these PAMPs include viral and bacterial infections and tissue necrosis. As the danger theory relates to pregnancy, these factors are not present during normal pregnancy, and thus the immune system is not activated excessively. However, in PE danger signals may be present leading to activation of their specific innate immune receptors, TLRs, excessive immune system activation, and inflammation via nuclear factor-κB (NF-κB) and interferons [14]–[20].

Bacterial components (LPS) acting on TLR2 and TLR4 have been implicated in the etiology of some forms of PE [21]–[23]; however whether viral/endogenous RNA plays a role in PE is unclear. Epidemiological studies have indicated that a primary viral infection during pregnancy is strongly associated with PE [24]–[27]. Excessive tissue necrosis and apoptosis from abnormal implantation or spiral artery remodeling at the feto-maternal interface may also cause the release of double-stranded RNA (dsRNA) and single-stranded (ssRNA) [28]–[31]. Therefore, we evaluated the roles of TLR3, which responds to dsRNA, and TLRs 7 and 8, which respond to ssRNA, in the development of PE. These TLRs are highly expressed in a variety of innate immune cells, vascular endothelial cells, and other cells at the feto-maternal interface including trophoblasts [31]–[35]. We hypothesized that activation of TLR3/7/8 is increased in placentas of women with PE and is sufficient to induce PE-like symptoms in mice.

## Results

### Placental TLR3/7/8 Activation and Inflammation in Women with PE

Placental tissue obtained from women delivering in 2010 was acquired from the Department of Pathology at Texas A&M Health Science Center/Scott & White Memorial Hospital. Anonymized patient characteristics were obtained in a retrospective manner and are presented in Table 1. Placental tissue and chart data were obtained for 13 pregnant (P) and 17 PE women diagnosed by the presence of hypertension and proteinuria. Some women in the pregnant (P) control group were clinically diagnosed with chorioamnionitis, chronic hypertension, proteinuria without hypertension, or premature rupture of membranes. There were no significant differences in age, body weight, or body mass index between the P and PE groups (Table 1). In addition to the hypertension and proteinuria, there was a significant decrease in gestational age, placental weight, and baby weight in PE women compared to P women (Table 1).

**Table 1:** Data for pregnant and preeclamptic patients.

	Pregnant (n = 13)	Preeclampsia (n = 17)
Age (years)	24.6±1.2	26.2±1.1
SBP (mm Hg)	132±7	162±5*
DBP (mm Hg)	71±4	94±2*
Weight (pounds)	195±21	191±8
BMI	34±4	33±2
Gestational Age (months)	37.8±0.7	30.7±1.1*
Placental weight (grams)	490±29	267±3*
Baby weight (grams)	2,886±186	1,549±225*

Data presented as mean ± SEM. SBP = systolic blood pressure, DBP = diastolic blood pressure, BMI = body mass index. *p<0.05 vs. pregnant.

**Figure 1:**
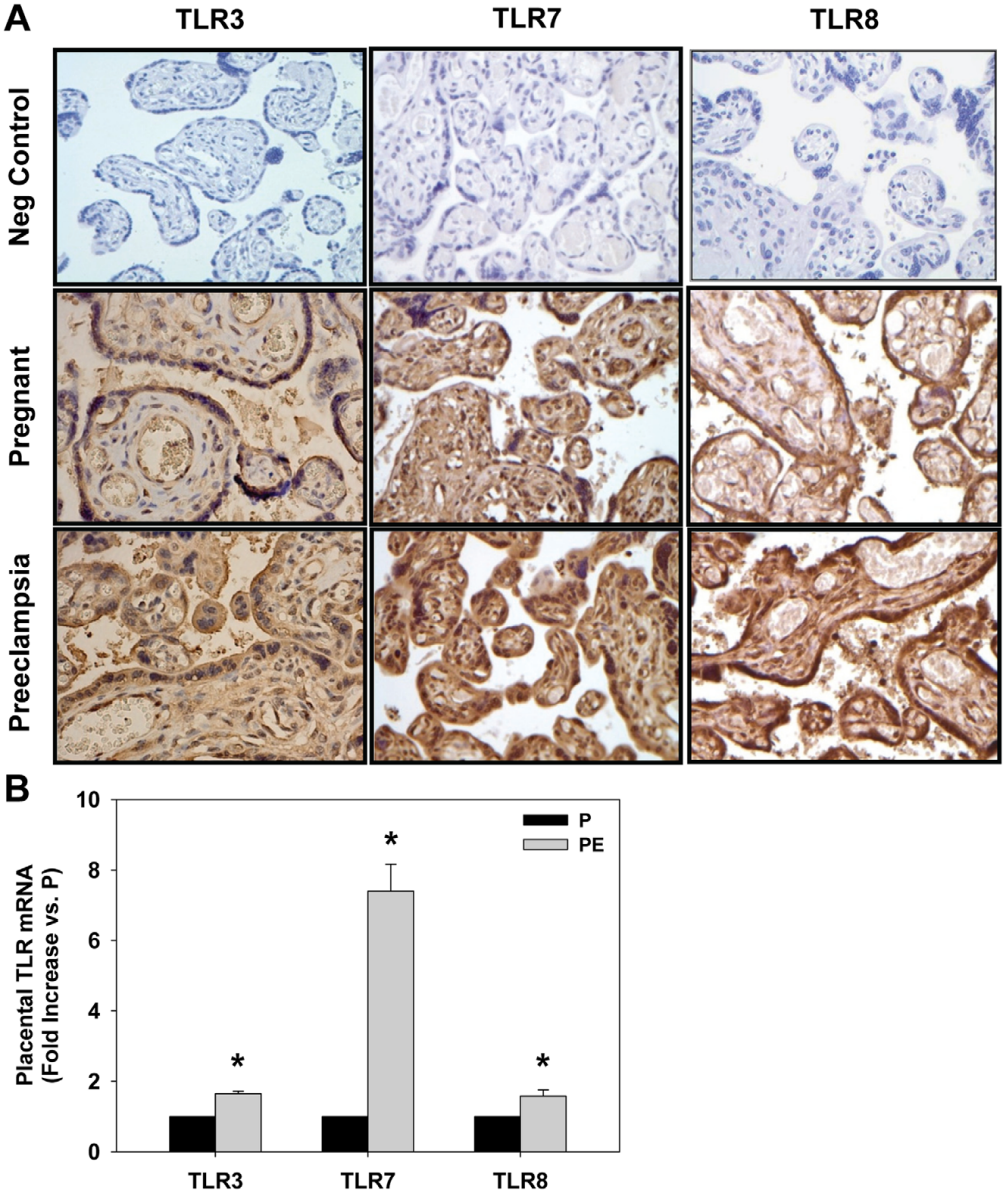
Increased TLR3/7/8 immunoreactivity and mRNA levels in placentas of women with preeclampsia. (A) Representative images where brown staining indicates immunoreactivity and blue indicates hematoxylin staining of nuclei in placentas from pregnant (P, n = 13, top panels) and preeclamptic (PE, n = 17, bottom panels) women. (B) mRNA levels of TLR3, TLR7, and TLR8 in placentas from PE and P women. Results are expressed as mean + SEM. *p<0.05 vs P.

To examine activation of TLR3/7/8 in placentas from P and PE women, we performed immunohistochemistry as well as measures of mRNA levels. Placentas from PE women had increased immunoreactivity of TLR3, TLR7, and TLR8 which was maximal in the outer syncytiotrophoblasts but was also present in the inner cytotrophoblasts (Figure 1A). Placental mRNA levels of TLR3, TLR7, and TLR8 were also increased significantly in women with PE compared to P women (Figure 1B).

### TLR3/7/8 Activation in Human Trophoblasts

**Figure 2:**
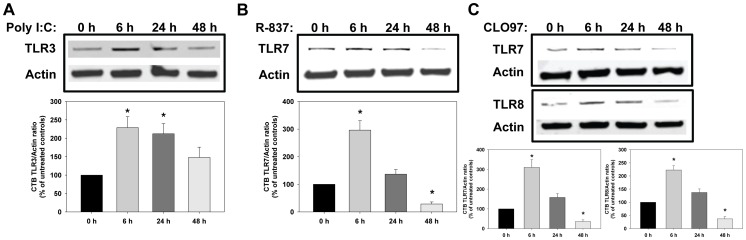
TLR3/7/8 activation in human trophoblasts. Representative images and densitometric analyses of TLR3, TLR7, and TLR8 protein levels from 4 independent experiments for (A) the TLR3 agonist poly I:C, (B) the TLR7 agonist R837, and (C) the TLR7/8 agonist CLO97. Results are expressed as mean + SEM. *p<0.05 vs baseline.

Next we determined whether direct activation of TLR3, TLR7, and TLR7/8 could occur in human cytotrophoblasts. Treatment of cytotrophoblasts with the TLR3-specific agonist poly I:C for 6, 24, and 48 hours significantly increased TLR3 protein levels at 6 and 24 hours which returned to baseline at 48 hours (Figure 2A). The TLR7-specific agonist R-837 significantly increased TLR7 protein levels ∼3-fold at 6 hours which returned to basal levels at 24 hours and decreased below basal levels at 48 hours (Figure 2B). CLO97, which activates both TLR7 and TLR8, significantly increased both TLR7 and TLR8 ∼2–3-fold at 6 hours while both returned to basal levels at 24 hours and decreased below basal levels at 48 hours (Figure 2C). Taken together, these data demonstrate that direct activation of TLR3/7/8 can occur in human trophoblasts, which leads to inflammation and immune cell activation via well-known TLR signaling pathways.

### TLR3/7/8 Activation in Pregnant Mice

We next examined whether TLR3 or TLR7/8 activation in mice could induce placental inflammation and PE-like symptoms. Pregnant mice were treated with either vehicle, poly I:C, R-837, or CLO97 given by intraperitoneal injection on gestational days 13, 15, and 17 followed by euthanization on gestational day 18. Eighty four inflammation-related genes were analyzed by quantitative Real-Time Polymerase Chain Reaction (qRT-PCR) and those that were significantly altered in all 3 groups are presented in Table 2. Consistent across all 3 groups of mice, the mRNA levels of 17 genes were increased significantly in placentas of mice following TLR3, TLR7, or TLR7/8 activation compared to vehicle-treated control mice and these included several pro-inflammatory chemokines, cytokines, receptor/ligands, and transcription factors (Table 2). The mRNA levels of only 2 genes were decreased significantly in the placentas of all 3 groups, *osteopontin* (*Spp1*) and *interleukin (IL)-18*.

**Table 2:** Inflammation-related genes significantly (p<0.05 vs. P) altered in placentas of mice treated with a TLR3, TLR7, or TLR7/8 agonist during pregnancy.

Gene Symbol	P vs. P+TLR3 (Fold change)	P vs. P+TLR7 (Fold change)	P vs. P+TLR7/8 (Fold change)
**Chemokines**			
*Ccl5 (RANTES)*	25.62±4.65	10.38±2.30	5.01±1.44
*Ccr3*	2.14±0.85	2.13±0.65	2.48±0.73
*Ccr10*	2.95±1.07	2.32±0.67	2.68±0.47
*Spp1*	−3.86±0.68	−3.29±0.68	−3.00±0.63
**Cytokines**			
*IL2*	2.27±0.04	2.67±0.34	2.23±0.71
*IL4ra*	4.25±0.72	5.18±1.21	4.22±0.63
*IL6*	2.21±0.16	2.72±0.16	2.83±0.63
*IL17A*	2.21±0.54	2.67±0.33	2.23±0.54
*IL18*	−2.09±0.58	−2.23±0.84	−2.38±0.26
*IL27*	2.81±0.44	3.81±0.71	2.87±0.71
*IFNg*	2.97±0.71	2.87±0.54	2.56±0.79
*TNF*	2.03±0.71	2.59±0.30	2.24±0.55
**Receptors/Ligands**			
*CD4*	3.07±0.40	4.98±0.53	5.74±1.12
*CD40*	2.06±0.55	5.40±0.65	4.08±0.96
*CD40ligand*	2.21±0.59	2.67±0.23	2.23±0.25
**Transcription Factors**			
*GATA3*	2.41±0.57	2.75±0.41	5.06±1.65
*Junb*	2.51±0.51	10.38±2.30	4.82±1.59
*NFkB*	4.68±0.65	4.02±0.48	4.46±0.85
*Stat4*	2.21±0.49	2.67±0.88	2.23±0.62

P = pregnant, P+TLR3 = pregnant+TLR3 agonist poly I:C, P+TLR7 = pregnant+TLR7 agonist R837, P+TLR7/8 = pregnant+TLR7/8 agonist CLO97. All p<0.05 vs. P, n>3 in each group.

**Figure 3:**
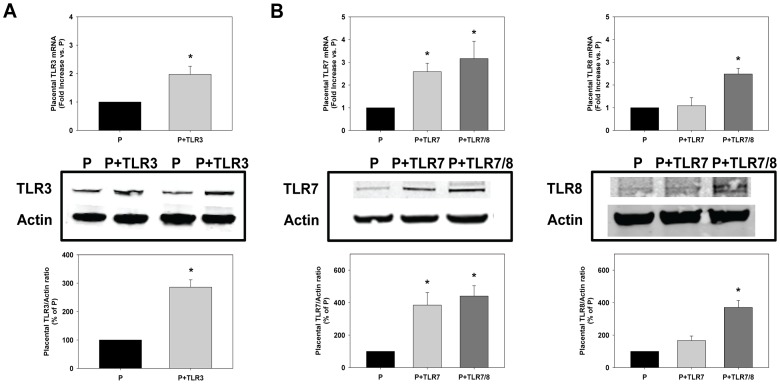
Placental TLR3/7/8 activation in pregnant mice. Fold-increase and representative images and densitometric analyses of TLR3, TLR7, and TLR8 mRNA and protein levels, respectively, from 4 independent experiments in mice treated with (A) the TLR3 agonist poly I:C and (B) the TLR7 agonist R837 and the TLR7/8 agonist CLO97. Results are expressed as mean + SEM. *p<0.05 vs P.

Treatment of pregnant mice with poly I:C via intraperitoneal injection lead to a significant increase in placental TLR3 gene and protein levels (Figure 3A). Treatment of pregnant mice with the TLR7-specific agonist R-837 significantly increased placental TLR7 mRNA and protein levels while having no effect on TLR8 expression (Figure 3B). Similarly, treatment of pregnant mice with the TLR7/8 agonist CLO97 significantly increased both TLR7 and TLR8 mRNA and protein levels (Figure 3B).

**Figure 4:**
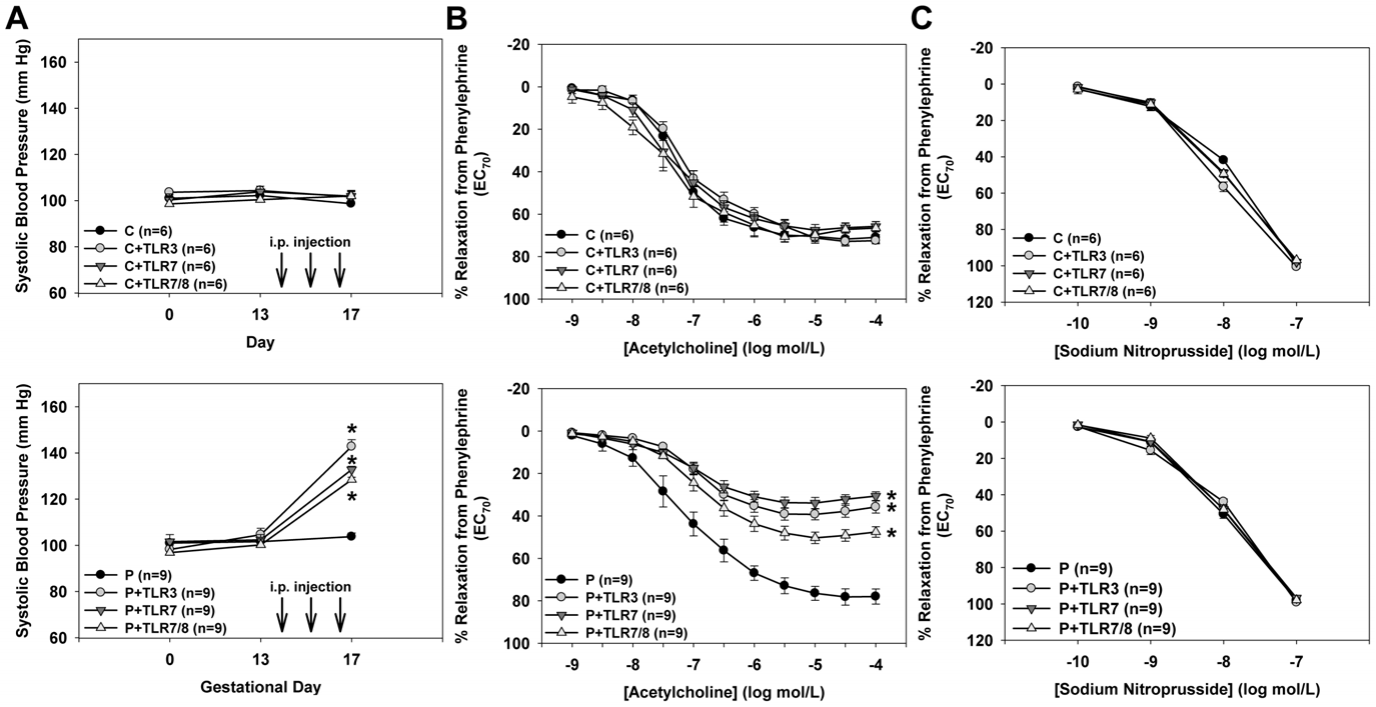
TLR3/7/8 activation in mice caused pregnancy-dependent hypertension and endothelial dysfunction. Measures of (A) systolic blood pressure, (B) aortic endothelium-dependent relaxation induced by acetylcholine, and (C) aortic endothelium-independent relaxation induced by sodium nitroprusside in non-pregnant (top panels) and pregnant (bottom panels) mice treated with TLR3 agonist poly I:C, the TLR7 agonist R837, or the TLR7/8 agonist CLO97. Results are expressed as mean ± SEM and the n is given in parentheses. *p<0.05 vs P.

We then determined whether TLR3 or TLR7/8 activation and subsequent placental inflammation was sufficient to induce PE-like symptoms in mice. In non-pregnant mice, activation of TLR3, TLR7, or TLR7/8 with poly I:C, R-837, or CLO97, respectively, had no effect on systolic blood pressure (Figure 4A). However, in pregnant mice these agonists all significantly increased systolic blood pressure at gestational day 17 compared to vehicle-treated controls (P: 104±2 mm Hg, P+TLR3: 143±3 mm Hg, P+TLR7: 133±1 mm Hg, P+TLR7/8: 129±1 mm Hg; all p<0.05 vs. P; Figure 4A). These data suggest that TLR3/7/8 activation causes hypertension only during pregnancy.

Next, we examined whether TLR3/7/8 activation in mice elicits endothelial dysfunction similar to that seen in women with PE. In non-pregnant mice, treatment with poly I:C, R-837, or CLO97 had no effects on aortic relaxation responses induced by acetylcholine (Figure 4B). However, TLR3, TLR7, or TLR7/8 activation during pregnancy caused a significant decrease in maximal aortic acetylcholine-induced relaxation responses (P: 78±4%, P+TLR3: 39±3%, P+TLR7: 34±3%, P+TLR7/8: 50±3%; all p<0.05 vs. P; Figure 4B). To determine whether TLR3 or TLR7/8 activation caused smooth muscle dysfunction, we measured aortic relaxation responses to the nitric oxide donor sodium nitroprusside. Figure 4C demonstrates that TLR3, TLR7, or TLR7/8 activation had no effect on sodium nitroprusside-induced aortic relaxation in either non-pregnant or pregnant mice. Taken together, these data show that TLR3/7/8 activation causes endothelial dysfunction only during pregnancy.

**Figure 5:**
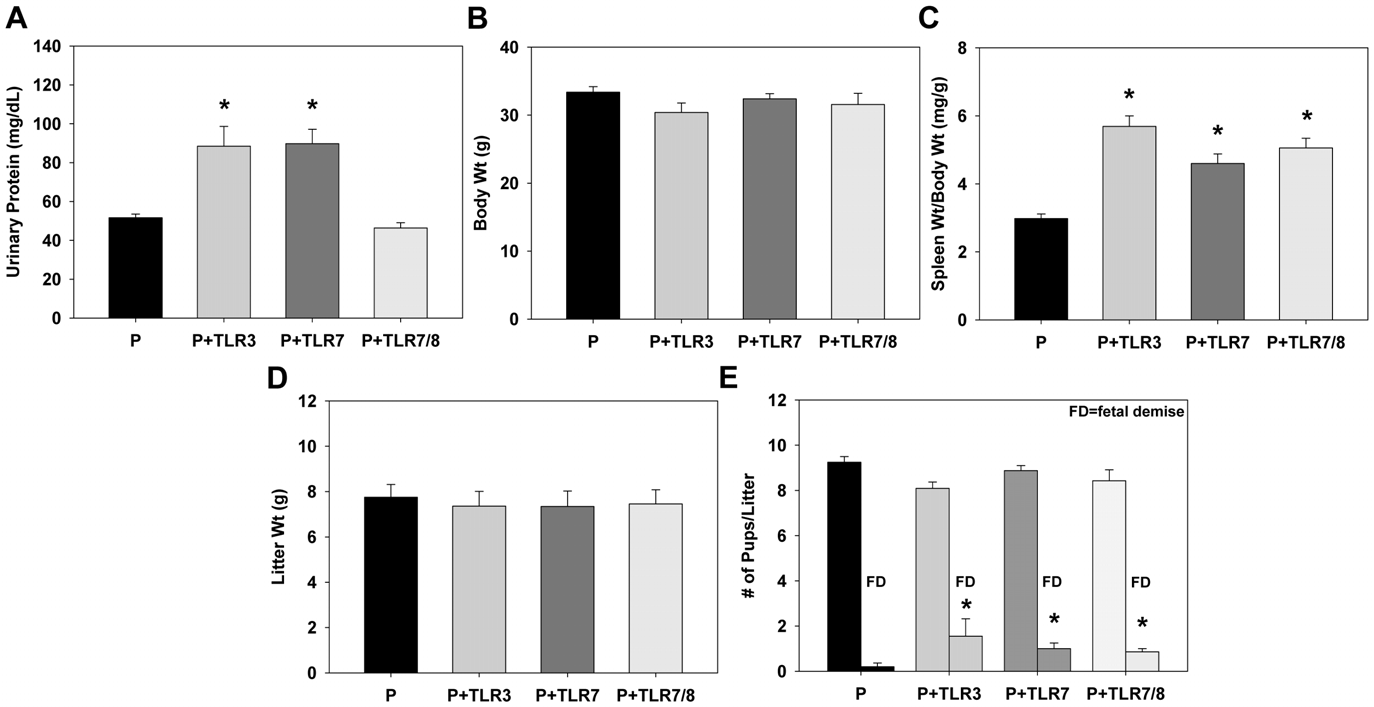
TLR3/7/8 activation in pregnant mice caused PE-like symptoms. Measures of (A) urinary protein concentration, (B) maternal body weight, (C) spleen weight/body weight, (D) litter weight, and (E) number of total pups/litter and fetal demise/litter in pregnant mice treated with the TLR3 agonist poly I:C, the TLR7 agonist R837, or the TLR7/8 agonist CLO97. Results are expressed as mean + SEM and n = 9 in each group. *p<0.05 vs. P.

In addition to hypertension and endothelial dysfunction, we examined whether TLR3/7/8 activation during pregnancy caused other PE-like symptoms. TLR3 or TLR7 activation with poly I:C or R-837 caused a significant increase in urinary protein excretion, however TLR7/8 activation with CLO97 unexpectedly had no effect (P: 52±2 mg/dL, P+TLR3: 89±10 mg/dL, P+TLR7: 90±7 mg/dL, P+TLR7/8: 46±3 mg/dL; Figure 5A). TLR3/7/8 activation during pregnancy had no effect on body weight (Figure 5B), however poly I:C, R-837, and CLO97 all caused splenomegaly, supporting a state of excessive inflammation and immune system activation during pregnancy (spleen weight/body weight-P: 3.0±0.1 mg/g, P+TLR3: 5.7±0.3 mg/g, P+TLR7: 4.6±0.3 mg/g, P+TLR7/8: 5.1±0.3 mg/g; all p<0.05 vs. P; Figure 5C).

Lastly, we determined the effects of TLR3/7/8 activation during pregnancy on fetal and placental development. Poly I:C, R837, or CLO97 had no effects on total litter weight (determined by weighing intact uterine horn containing pups, placentas, and amniotic fluid) which were ∼7.5 g in all 4 groups (Figure 5D) or on the number of pups/litter which were all around ∼8–9 pups/litter (Figure 5E). However, poly I:C, R-837, and CLO97 all caused a significant increase in fetal demise assessed as the mean number of malformed or resorbed pups/litter on day 18 of pregnancy (P: 0.2±0.2, P+TLR3: 1.6±0.8, P+TLR7: 1.0±0.3, P+TLR7/8: 0.9±0.1; all p<0.05 vs. P; Figure 5E).

## Discussion

The initiating factors that cause PE in ∼10% of all pregnancies remain elusive however placental dysfunction and inflammation play key roles. Here we present a unifying hypothesis that these, in some women, are caused by RNA-mediated activation of placental TLR3 and TLR7/8 leading to PE. Supportive evidence comes from our findings that TLR3/7/8 immunoreactivity and mRNA levels are increased significantly in placentas of women with PE, that direct activation of TLR3/7/8 occurs in human trophoblasts, and that TLR3/7/8 activation in mice causes placental inflammation, splenomegaly, fetal demise, and pregnancy-dependent hypertension and endothelial dysfunction.

Women diagnosed with PE had a significant increase in placental immunoreactivity and mRNA levels of TLR3, TLR7, and TLR8 compared to normotensive women. A previous study also reported increased TLR3 staining in the trophoblast, vascular endothelium, and stroma of PE women, however TLR7 and TLR8 staining were not examined [36]. Even though our normotensive P group consisted of women with chorioamnionitis, chronic hypertension, proteinuria without hypertension, or premature rupture of membranes, there were no placentas in this group that had increased levels of TLR3. In the P group, only 3/13 placentas for TLR7 and 6/13 placentas for TLR8 exhibited increased levels of these receptors. There was no apparent trend between significant placental TLR activation and clinical indication in the P group. For instance, of the 6 women with chorioamnionitis none had increased levels of TLR3, only 1 had increased levels of TLR7, and 2 for TLR8. Interestingly, 2 women had increased levels for both TLR7 and TLR8 but not TLR3. One woman was a smoker having her 3^rd^ child and the other experienced pre-term rupture of membranes which may have resulted in an acute release of ssRNA. In the PE group, only 4/17 placentas for TLR3 and 2/17 placentas for TLR7 had decreased levels of these receptors while no placentas from PE women had decreased levels of TLR8. These data suggest that significant placental activation of TLR3, TLR7, and TLR8 together is highly associated with PE, but whether these occur prior to the development of PE in women remains to be determined.

Trophoblasts are the main cell type at the feto-maternal interface and exert many functions necessary for normal pregnancy including the regulation of spiral artery remodeling and angiogenesis as well as pathogen sensing and immune system signaling. Trophoblasts express numerous TLRs including TLR3, TLR7, and TLR8 and are able to secrete NF-κB-derived pro-inflammatory chemokines and cytokines [19], [31], [32], [37]–[41]. To determine whether TLR3/7/8 activation occurs in trophoblasts, we treated human trophoblasts with agonists for TLR3, TLR7, or TLR7/8. All 3 agonists were able to rapidly increase protein levels of their respective TLR with the peak being 6 hours. Together these findings suggest that persistent TLR3/7/8 activation (i.e., chronic elevated levels of dsRNA and ssRNA) in trophoblasts may be the cause of the placental dysfunction and inflammation seen in PE.

If placental TLR3/7/8 activation is sufficient to cause PE, then treatment of mice with TLR3/7/8 agonists should elicit endothelial dysfunction, proteinuria, and hypertension only if the animal is pregnant. We have previously reported that TLR3 activation with poly I:C in rats and mice causes systemic inflammation in both pregnant and non-pregnant animals, however only pregnant animals developed hypertension, proteinuria, and endothelial dysfunction [42]–[44]. In the current study, we confirm these findings.

Additionally, 19 placental genes that are significantly altered in poly I:C-treated mice are also significantly altered in mice treated with either a TLR7 or TLR7/8 agonist. The 17 placental genes that were significantly increased in all 3 groups are known to play key roles in the inflammatory response. PE women have elevated levels of interferon-γ, tumor necrosis factor-α, IL-17A, IL-6, and CD-40/CD-40 ligand [45]–[47], all genes that were significantly increased in the placentas of TLR3, TLR7, and TLR7/8 agonist-treated mice. The 2 placental genes that were decreased in all 3 groups interestingly have pro-inflammatory chemokine/cytokine roles. However, osteopontin, which increases throughout normal gestation, is also an extracellular matrix protein that plays a role in cell-cell and cell-matrix interactions [48]. It is possible that this decrease in placental osteopontin levels may contribute to the disorganization of placental cells evident in PE [49]. Additionally, osteopontin and IL-18, the other placental gene decreased in TLR3/7/8 agonist-treated mice, both potentiate T helper cell type 1 immune responses [48], whereas IL-2, IL-6, and IL-17A, all significantly increased in placentas of TLR3/7/8 agonist-treated mice, potentiate a T helper cell type 17 immune response characteristic of PE [46], [47]. More studies are needed to determine the temporal and functional effects of altered placental gene expression including the placental downregulation of osteopontin and IL-18 and their role in the development of PE.

Similar to TLR3 activation, TLR7/8 activation during pregnancy in mice caused pregnancy-dependent hypertension, endothelial dysfunction, splenomegaly, and an increased incidence of fetal demise. Importantly, blood pressure and endothelial function were not affected by TLR7 or TLR7/8 activation in non-pregnant mice. These findings demonstrate that placental activation of TLR3/7/8 is crucial for the development of endothelial dysfunction and hypertension during pregnancy. Fetal demise in mice likely results from placental dysfunction and decreased placental perfusion and somewhat mimics intra-uterine growth restriction, commonly seen in women with PE. We reported that TLR3 ligation in mice increased the incidence of fetal demise, and almost identical results were seen in TLR7 as well as TLR7/8 agonist-treated mice. A previous study found that rats treated with the TLR4 agonist lipopolysaccharide at gestational day 14.5 also exhibited increased fetal demise as a result of decreased placental perfusion [50]. Although TLR3 and TLR7 activation induced proteinuria, TLR7/8 activation did not. Some studies have demonstrated that not only is TLR8 functional in mice, but that it may suppress TLR7-mediated effects. Demaria and colleagues reported that *Tlr8^−/−^* mice develop autoimmune glomerulonephritis due to excessive TLR7 expression and activation, and that this was attenuated in double *Tlr7^−/−/^Tlr8^−/−^* mice [51]. Unfortunately, urinary protein concentrations were not measured, but their findings together with our results suggest that the stimulation of renal, not placental, TLR8 may have a protective effect on renal function. Nonetheless, excessive activation of placental TLR7/8 is sufficient to induce pregnancy-dependent hypertension and endothelial dysfunction similar to that induced by TLR3 activation. However, examination of the role of TLR8 signaling in the mouse and how this might contribute to the development of PE-like symptoms is needed. If TLR8 signaling in mice is found to be non-functional, then it is likely that the mechanisms that cause PE in CLO97-treated mice are due to TLR7 activation, similar to that of R837-treated mice.

Placental activation of TLR3/7/8 by dsRNA and ssRNA could arise from latent viruses, viruses acquired during gestation, as well as excessive cellular necrosis/apoptosis resulting from aberrant implantation, placentation, placental hypoxia, and/or trophoblast invasion [28], [30]. Additionally, heterologous RNA released from necrotic cells or associated with in vitro transcription was reported to be an endogenous ligand for TLR3 [52]. While extracellular heterologous RNA levels are normally low, high levels of extracellular heterologous RNA are evident at sites of inflammation and tissue necrosis, similar to that of dsRNA and ssRNA [52]. Whether heterologous RNA can also activate TLR7/8 is unknown currently. While TLR3 signals through Toll-IL-1 receptor-domain-containing adaptor inducing interferon-β (TRIF), TLR7 and TLR8 signal through myeloid differentiation marker 88 (MyD88). However, both pathways converge to activate NF-κB leading to the transcription of numerous pro-inflammatory chemokines and cytokines. Inflammation is necessary for placental and fetal development, however excessive inflammation resulting from persistent placental TLR3/7/8 activation may explain the increase in circulating pro-inflammatory cytokines evident in women with PE.

Here we demonstrate that TLR3, TLR7, and TLR8 levels are significantly increased in placentas from PE women at delivery, but it is unknown if this occurs prior to the onset of PE symptoms in women. Given our findings that TLR3, TLR7, or TLR7/8 activation was sufficient to produce pregnancy-dependent hypertension and endothelial dysfunction in mice, we hypothesize that excessive TLR3/7/8 activation initiates the symptoms of PE in women, however prospective studies and earlier measures are needed. Our results suggest that TLR3, TLR7, and TLR8 play a significant role in the development of PE and may be potential therapeutic targets to diminish the severity of PE symptoms in women.

## Materials and Methods

### Ethics Statement

The research study involving human placental samples was reviewed by the Texas A&M Health Science Center/Scott and White Institutional Review Board (Federal Assurance #FWA00003358, IRB Registration #IRB00000706) prior to initiation, and all patient identifiers were removed from sample and patient data. The IRB approved the study as exempt from requiring patient consent according to category 45 CFR 46.101(b)(4) (Project # 100581, 12/27/2010) and the samples were available from an archive maintained by the Department of Pathology for research purposes. All procedures performed in mice were approved by the Texas A&M Health Science Center/Scott and White Memorial Hospital IACUC in accordance with the *NIH Guide for the Care and Use and Care of Laboratory Animals*.

### Patient Data and Human Placental Immunohistochemistry and mRNA Levels

Paraffin-embedded blocks of placental tissue were obtained from P (n = 13) and PE (n = 17) women who delivered between March and September 2010 by the Texas A&M Health Science Center/Scott and White Memorial Hospital Department of Pathology. Twenty slides from each block (5 µm thick) were used for immunohistochemistry. The samples were first deparaffinized and then antigen retrieval was performed. This was followed by quenching of endogenous peroxidase using 3% hydrogen peroxide for 5 minutes. The slides were then blocked with appropriate diluted blocking serum for 20 minutes and were stained with antibodies using the following concentrations: TLR3 at 1∶100 (Imgenex; San Diego, CA), TLR7 at 1∶500 (Imgenex), and TLR8 at 1∶500 (Imgenex) for 1 hour at room temperature. Incubation with biotinylated secondary antibody for 45 minutes was followed by streptavidin-peroxidase for 10 minutes, and then developed with 3,3′-diaminobenzidine (DAB). Hematoxylin was used as a counterstain (blue) to denote cell nuclei and slides incubated with secondary antibody only served as negative controls. Additionally, formalin-fixed, paraffin-embedded sections of placental tissue (10 µm thick) were obtained from both P and PE patients for qRT-PCR reactions. First, paraffin was removed with deparaffinization solution by incubating at 56°C for 3 minutes followed by slow cooling to room temperature. RNA was isolated using a RNeasy FFPE kit (Qiagen; Valencia, CA) following the manufacturer’s protocol. cDNA was prepared from 500 ηg RNA and was further pre-amplified using a RT^2^ PreAmp cDNA synthesis kit (Qiagen) per the manufacturer’s protocol. Human primers for TLR3 (SABiosciences), TLR7 (SABiosciences), and TLR8 (Origene) were used for qRT-PCR and were performed using SYBR Green (SABiosciences, Valencia, CA). Amplification of the genes of interest and the housekeeping control gene GAPDH was done in duplicate from each sample and a no template control. The relative fold change in expression level compared to placentas from P women was determined using the 2 ^(−ΔΔCt)^ method.

### Tissue Culture

Human SGHPL-4 cytotrophoblasts [28], [53], obtained from Professor Guy Whitley (St. George’s, University of London, UK), were cultured in Ham’s F-10 media supplemented with 10% fetal bovine serum, penicillin G (100 units/ml), streptomycin (100 mg/ml) and 2 mM l-glutamine. Cells were incubated at 37°C in a 5% CO_2_ incubator and serum starved for 24 hours prior to treatment with either vehicle, poly I:C (2 µg/ml), R837 (10 µg/ml), or CL097 (25 µg/ml). Cells were lysed after 6, 24, or 48 hours of treatment and protein was isolated subsequently for the immunoblotting studies described below.

### Mouse Studies

Male C57BL/6J mice, used for mating purposes only, and female C57BL/6J mice were purchased from Jackson Laboratories (Bar Harbor, ME). Female mice, aged 10–12 weeks, were grouped as pregnant (P), pregnant treated with poly I:C (P+TLR3), pregnant treated with R837 (P+TLR7), or pregnant treated with CL097 (P+TLR7/8). Non-pregnant mice served as controls for each of the 4 groups (C, C+TLR3, C+TLR7, C+TLR7/8). P mice were treated with vehicle (phosphate buffered saline), P+TLR3 mice were treated with poly I:C (20 mg/kg), P+TLR7 mice were treated with R837 (4 mg/kg), and P+TLR7/8 mice were treated with CL097 (20 mg/kg) via intraperitoneal injections on days 13, 15, and 17 of gestation while non-pregnant mice were given the same injections on corresponding days. Tail-cuff systolic blood pressures (IITC, Inc.; Woodland Hills, CA) were measured at baseline and on day 13 and day 17, prior to injections, as described previously [42]. Mice were weighed and then sacrificed on day 18 of pregnancy or corresponding day in non-pregnant mice and blood, urine, and tissues were collected. Spleens were excised and weighed and the spleen weight/body weight ratio (mg/g) was calculated. The entire intact uterine horn containing pups, placentas, and amniotic fluid was excised and weighed. Pups and placentas were then dissected out and counted, and the number of pups experiencing fetal demise due to resorption or malformations was also counted. Protein concentrations in urine obtained on day 18 were measured using the pyrogallol red method (Total Protein Kit, Micro Pyrogallol Red Method, Sigma) as described previously [42]. Relaxation responses were assessed in isolated endothelium-intact aortic rings (2 mm) obtained from mice on day 18 as described previously [42]. Briefly, aortas were mounted on stainless steel pins in a myograph (Danish Myo Technology; Ann Arbor, MI) and were warmed to 37°C, oxygenated with 95% O_2_/5% CO_2_, and set at a passive tension of 0.75 g of force. Following examination of viability, vessels were contracted with an EC_70_ concentration of phenylephrine (1 µM) followed by cumulative concentrations of the endothelium-dependent dilator acetylcholine. Vessels were then washed until passive tension returned to baseline followed by phenylephrine-induced contraction and the addition of cumulative concentrations of the endothelium-independent dilator sodium nitroprusside. Concentrations-response curves were generated based on the percent of relaxation from the phenylephrine-induced contraction.

### Immunoblotting

Human trophoblasts and mouse placentas were lysed/homogenized in the presence of protease and phosphatase inhibitors in lysis buffer (Cell Signaling; Boston, MA). Electrophoresis was performed on 4–12% sodium dodecyl sulfate polyacrylamide gels and transferred onto nitrocellulose membranes (Millipore; Billerica, MA). Western blot analyses were performed as described previously [42] using the following primary antibodies: TLR3 1∶1,000 (Imgenex, San Diego, CA), TLR7 1∶500 (Ebioscience; San Diego, CA), and TLR8 1∶500 (Imgenex), and beta-actin 1∶10,000 (Sigma; St. Louis, MO). The secondary antibodies consisted of anti-rabbit and anti-mouse IgGs conjugated to Alexa-Fluor 680 or IRDye 800 (LI-COR Biosciences; Lincoln, NE). Infrared visualization was used followed by densitometric analyses using the provided software (Odyssey System, LI-COR Biosciences). The ratio of the densitometry of the TLR band to the actin band was calculated and then expressed as a % of untreated control trophoblasts or P mice.

### qRT-PCR

RNA from mouse placentas was isolated using an RNEasy Kit (Qiagen; Valencia, CA). An iScript Synthesis Kit (Bio-Rad; Hercules, CA) was used to perform reverse transcription using 1 µg total RNA to prepare cDNA, which was analyzed via qRT-PCR reactions performed using a SYBR Green System (Qiagen) per the manufacturer’s instructions. The RT^2^ Profiler Th1-Th2-Th3 PCRarray from SABiosciences (Qiagen) was used and consisted of 84 genes involved in immune cell activation. The 2 ^(−ΔΔCt)^ method was used to determine the fold change in RNA expression compared to placentas from P mice.

### Statistical Analyses

Results are presented as mean ± standard error of the mean. The Student’s t-test was used to compare measures between P and PE women. For measurements examined in human trophoblasts and pregnant mice, a one-way analysis of variance was used followed by the Student’s-Newman-Keuls *post hoc* test when necessary. Temporal changes in mice for systolic blood pressure were analyzed by a repeated-measures analysis of variance followed by the Student’s-Newman-Keuls *post hoc* test when necessary. The significance level was 0.05.

## References

[1] Jonsdottir LS, Arngrimsson R, Geirsson RT, Sigvaldason H, Sigfusson N (1995) Death rates from ischemic heart disease in women with a history of hypertension in pregnancy. Acta Obstet Gynecol Scand 74: 772–776. 8533558 10.3109/00016349509021195

[2] Irgens HU, Reisaeter L, Irgens LM, Lie RT (2001) Long term mortality of mothers and fathers after pre-eclampsia: population based cohort study. BMJ 323: 1213–1217. 11719411 10.1136/bmj.323.7323.1213 PMC59993

[3] Smith GC, Pell JP, Walsh D (2001) Pregnancy complications and maternal risk of ischaemic heart disease: a retrospective cohort study of 129,290 births. Lancet 357: 2002–2006. 11438131 10.1016/S0140-6736(00)05112-6

[4] Wilson BJ, Watson MS, Prescott GJ, Sunderland S, Campbell DM, et al (2003) Hypertensive diseases of pregnancy and risk of hypertension and stroke in later life: results from cohort study. BMJ 326: 845. 12702615 10.1136/bmj.326.7394.845 PMC153466

[5] Schiessl B (2007) Inflammatory response in preeclampsia. Mol Aspects Med 28: 210–219. 17532463 10.1016/j.mam.2007.04.004

[6] Bonney EA (2007) Preeclampsia: a view through the danger model. J Reprod Immunol 76: 68–74. 17482268 10.1016/j.jri.2007.03.006 PMC2246056

[7] Saito S, Sakai M, Sasaki Y, Nakashima A, Shiozaki A (2007) Inadequate tolerance induction may induce pre-eclampsia. J Reprod Immunol 76: 30–39. 17935792 10.1016/j.jri.2007.08.002

[8] Saito S, Shiozaki A, Nakashima A, Sakai M, Sasaki Y (2007) The role of the immune system in preeclampsia. Mol Aspects Med 28: 192–209. 17433431 10.1016/j.mam.2007.02.006

[9] Redman CW, Sacks GP, Sargent IL (1999) Preeclampsia: an excessive maternal inflammatory response to pregnancy. Am J Obstet Gynecol 180: 499–506. 9988826 10.1016/s0002-9378(99)70239-5

[10] Visser N, van Rijn BB, Rijkers GT, Franx A, Bruinse HW (2007) Inflammatory changes in preeclampsia: current understanding of the maternal innate and adaptive immune response. Obstet Gynecol Surv 62: 191–201. 17306041 10.1097/01.ogx.0000256779.06275.c4

[11] Sibai BM (1991) Immunologic aspects of preeclampsia. Clin Obstet Gynecol 34: 27–34. 2025973

[12] Schneider K, Knutson F, Tamsen L, Sjoberg O (1994) HLA antigen sharing in preeclampsia. Gynecol Obstet Invest 37: 87–90. 8150376 10.1159/000292531

[13] Dempsey JC, Sorensen TK, Qiu CF, Luthy DA, Williams MA (2003) History of abortion and subsequent risk of preeclampsia. J Reprod Med 48: 509–514. 12953325

[14] Hubel CA (1999) Oxidative stress in the pathogenesis of preeclampsia. Proc Soc Exp Biol Med 222: 222–235. 10601881 10.1177/153537029922200305

[15] Levine RJ, Lam C, Qian C, Yu KF, Maynard SE, et al (2006) Soluble endoglin and other circulating antiangiogenic factors in preeclampsia. N Engl J Med 355: 992–1005. 16957146 10.1056/NEJMoa055352

[16] Paine MA, Scioscia M, Gumaa KA, Rodeck CH, Rademacher TW (2006) P-type inositol phosphoglycans in serum and amniotic fluid in active pre-eclampsia. J Reprod Immunol 69: 165–179. 16384607 10.1016/j.jri.2005.09.008

[17] Koga K, Cardenas I, Aldo P, Abrahams VM, Peng B, et al (2009) Activation of TLR3 in the trophoblast is associated with preterm delivery. Am J Reprod Immunol 61: 196–212. 19239422 10.1111/j.1600-0897.2008.00682.x PMC2765929

[18] Koga K, Mor G (2010) Toll-like receptors at the maternal-fetal interface in normal pregnancy and pregnancy disorders. Am J Reprod Immunol 63: 587–600. 20367625 10.1111/j.1600-0897.2010.00848.x PMC3025804

[19] Mor G, Romero R, Aldo PB, Abrahams VM (2005) Is the trophoblast an immune regulator? The role of Toll-like receptors during pregnancy. Crit Rev Immunol 25: 375–388. 16167887 10.1615/critrevimmunol.v25.i5.30

[20] Luppi P, Tse H, Lain KY, Markovic N, Piganelli JD, et al (2006) Preeclampsia activates circulating immune cells with engagement of the NF-kappaB pathway. Am J Reprod Immunol 56: 135–144. 16836616 10.1111/j.1600-0897.2006.00386.x

[21] Xie F, Hu Y, Turvey SE, Magee LA, Brunham RM, et al (2010) Toll-like receptors 2 and 4 and the cryopyrin inflammasome in normal pregnancy and pre-eclampsia. BJOG 117: 99–108. 20002372 10.1111/j.1471-0528.2009.02428.x

[22] Riley JK, Nelson DM (2010) Toll-like receptors in pregnancy disorders and placental dysfunction. Clin Rev Allergy Immunol 39: 185–193. 19866377 10.1007/s12016-009-8178-2

[23] Kim YM, Romero R, Oh SY, Kim CJ, Kilburn BA, et al (2005) Toll-like receptor 4: a potential link between “danger signals,” the innate immune system, and preeclampsia? Am J Obstet Gynecol 193: 921–927. 16157088 10.1016/j.ajog.2005.07.076

[24] Trogstad LI, Eskild A, Bruu AL, Jeansson S, Jenum PA (2001) Is preeclampsia an infectious disease? Acta Obstet Gynecol Scand 80: 1036–1038. 11703202 10.1034/j.1600-0412.2001.801112.x

[25] Conde-Agudelo A, Villar J, Lindheimer M (2008) Maternal infection and risk of preeclampsia: systematic review and metaanalysis. Am J Obstet Gynecol 198: 7–22. 18166297 10.1016/j.ajog.2007.07.040

[26] Rustveld LO, Kelsey SF, Sharma R (2008) Association between maternal infections and preeclampsia: a systematic review of epidemiologic studies. Matern Child Health J 12: 223–242. 17577649 10.1007/s10995-007-0224-1

[27] Arechavaleta-Velasco F, Ma Y, Zhang J, McGrath CM, Parry S (2006) Adeno-associated virus-2 (AAV-2) causes trophoblast dysfunction, and placental AAV-2 infection is associated with preeclampsia. Am J Pathol 168: 1951–1959. 16723710 10.2353/ajpath.2006.050781 PMC1606626

[28] LaMarca HL, Nelson AB, Scandurro AB, Whitley GS, Morris CA (2006) Human cytomegalovirus-induced inhibition of cytotrophoblast invasion in a first trimester extravillous cytotrophoblast cell line. Placenta 27: 137–147. 15921739 10.1016/j.placenta.2005.03.003

[29] Whitley GS, Cartwright JE (2010) Cellular and molecular regulation of spiral artery remodelling: lessons from the cardiovascular field. Placenta 31: 465–474. 20359743 10.1016/j.placenta.2010.03.002 PMC2882556

[30] Whitley GS, Dash PR, Ayling LJ, Prefumo F, Thilaganathan B, et al (2007) Increased apoptosis in first trimester extravillous trophoblasts from pregnancies at higher risk of developing preeclampsia. Am J Pathol 170: 1903–1909. 17525258 10.2353/ajpath.2007.070006 PMC1899436

[31] Aldo PB, Mulla MJ, Romero R, Mor G, Abrahams VM (2010) Viral ssRNA induces first trimester trophoblast apoptosis through an inflammatory mechanism. Am J Reprod Immunol 64: 27–37. 20175771 10.1111/j.1600-0897.2010.00817.x PMC2889030

[32] Gonzalez JM, Xu H, Ofori E, Elovitz MA (2007) Toll-like receptors in the uterus, cervix, and placenta: is pregnancy an immunosuppressed state? Am J Obstet Gynecol 197: e291–296. 10.1016/j.ajog.2007.06.021 17826427

[33] Luppi P, Deloia JA (2006) Monocytes of preeclamptic women spontaneously synthesize pro-inflammatory cytokines. Clin Immunol 118: 268–275. 16337193 10.1016/j.clim.2005.11.001

[34] Zimmer S, Steinmetz M, Asdonk T, Motz I, Coch C, et al (2011) Activation of endothelial toll-like receptor 3 impairs endothelial function. Circ Res 108: 1358–1366. 21493895 10.1161/CIRCRESAHA.111.243246

[35] Patni S, Wynen LP, Seager AL, Morgan G, White JO, et al (2009) Expression and activity of Toll-like receptors 1–9 in the human term placenta and changes associated with labor at term. Biol Reprod 80: 243–248. 18815357 10.1095/biolreprod.108.069252

[36] Pineda A, Verdin-Teran SL, Camacho A, Moreno-Fierros L (2011) Expression of toll-like receptor TLR-2, TLR-3, TLR-4 and TLR-9 is increased in placentas from patients with preeclampsia. Arch Med Res 42: 382–391. 21843566 10.1016/j.arcmed.2011.08.003

[37] Abrahams VM (2008) Pattern recognition at the maternal-fetal interface. Immunol Invest 37: 427–447. 18716932 10.1080/08820130802191599

[38] Abrahams VM, Mor G (2005) Toll-like receptors and their role in the trophoblast. Placenta 26: 540–547. 15993703 10.1016/j.placenta.2004.08.010

[39] Abrahams VM, Schaefer TM, Fahey JV, Visintin I, Wright JA, et al (2006) Expression and secretion of antiviral factors by trophoblast cells following stimulation by the TLR-3 agonist, Poly(I : C). Hum Reprod 21: 2432–2439. 16751646 10.1093/humrep/del178

[40] Abrahams VM, Visintin I, Aldo PB, Guller S, Romero R, et al (2005) A role for TLRs in the regulation of immune cell migration by first trimester trophoblast cells. J Immunol 175: 8096–8104. 16339547 10.4049/jimmunol.175.12.8096

[41] Krikun G, Lockwood CJ, Abrahams VM, Mor G, Paidas M, et al (2007) Expression of Toll-like receptors in the human decidua. Histol Histopathol 22: 847–854. 17503341 10.14670/HH-22.847

[42] Chatterjee P, Chiasson VL, Kopriva SE, Young KJ, Chatterjee V, et al (2011) Interleukin 10 deficiency exacerbates toll-like receptor 3-induced preeclampsia-like symptoms in mice. Hypertension 58: 489–496. 21768525 10.1161/HYPERTENSIONAHA.111.172114

[43] Chatterjee P, Weaver LE, Chiasson VL, Young KJ, Mitchell BM (2011) Do double-stranded RNA receptors play a role in preeclampsia? Placenta 32: 201–205. 21292321 10.1016/j.placenta.2010.12.026

[44] Tinsley JH, Chiasson VL, Mahajan A, Young KJ, Mitchell BM (2009) Toll-like receptor 3 activation during pregnancy elicits preeclampsia-like symptoms in rats. Am J Hypertens 22: 1314–1319. 19779466 10.1038/ajh.2009.185

[45] Jonsson Y, Ruber M, Matthiesen L, Berg G, Nieminen K, et al (2006) Cytokine mapping of sera from women with preeclampsia and normal pregnancies. J Reprod Immunol 70: 83–91. 16388854 10.1016/j.jri.2005.10.007

[46] Toldi G, Rigo J Jr, Stenczer B, Vasarhelyi B, Molvarec A (2011) Increased prevalence of IL-17-producing peripheral blood lymphocytes in pre-eclampsia. Am J Reprod Immunol 66: 223–229. 21306467 10.1111/j.1600-0897.2011.00987.x

[47] Santner-Nanan B, Peek MJ, Khanam R, Richarts L, Zhu E, et al (2009) Systemic increase in the ratio between Foxp3+ and IL-17-producing CD4+ T cells in healthy pregnancy but not in preeclampsia. J Immunol 183: 7023–7030. 19915051 10.4049/jimmunol.0901154

[48] Johnson GA, Burghardt RC, Bazer FW, Spencer TE (2003) Osteopontin: roles in implantation and placentation. Biol Reprod 69: 1458–1471. 12890718 10.1095/biolreprod.103.020651

[49] Xia J, Qiao F, Su F, Liu H (2009) Implication of expression of osteopontin and its receptor integrin alphanubeta3 in the placenta in the development of preeclampsia. J Huazhong Univ Sci Technolog Med Sci 29: 755–760. 20037822 10.1007/s11596-009-0617-z

[50] Renaud SJ, Cotechini T, Quirt JS, Macdonald-Goodfellow SK, Othman M, et al (2011) Spontaneous pregnancy loss mediated by abnormal maternal inflammation in rats is linked to deficient uteroplacental perfusion. J Immunol 186: 1799–1808. 21187445 10.4049/jimmunol.1002679

[51] Demaria O, Pagni PP, Traub S, de Gassart A, Branzk N, et al (2010) TLR8 deficiency leads to autoimmunity in mice. J Clin Invest 120: 3651–3662. 20811154 10.1172/JCI42081 PMC2947223

[52] Kariko K, Ni H, Capodici J, Lamphier M, Weissman D (2004) mRNA is an endogenous ligand for Toll-like receptor 3. J Biol Chem 279: 12542–12550. 14729660 10.1074/jbc.M310175200

[53] Whitley GS (2006) Production of human trophoblast cell lines. Methods Mol Med 121: 219–228. 16251746 10.1385/1-59259-983-4:217

